# Bibliography

**Published:** 1899-10

**Authors:** 


### Bibliography.

Interstitial Gingivitis; or, So-called Pyorrhœa Alveolaris. By Eugene S. Talbot, M.D., D.D.S., Professor of Dental and Oral Surgery, Northwestern University, Woman's Medical School; Honorary President of the Dental Section of the Tenth International Medical Congress, etc. With Seventy-three Illustrations. The S. S. White Dental Manufacturing Company, Philadelphia, 1899.

The disease of the oral cavity, known as pyorrhœa alveolaris, has received more extended examination than any of the pathological conditions coming under the care of the dentist, and yet, at the present time, we are afflicted with more diverse sentiments as to its etiology and treatment than with any other lesion affecting the gingival border, and, apparently, we are as far from arriving at a decisive opinion as were the writers of the last century.

It is, therefore, with peculiar satisfaction that this work of Dr. Talbot is welcomed, for whatever opinions may be held as to his conclusions, no criticism can justly be made upon the methods pursued. These have been in the direction of true scientific research, evolving no theory unless based on demonstrated facts.

The labor and expense devoted to this should, and doubtless will, receive the highest commendation. The author has endeavored to eliminate his personal opinions by "having researches made by more than one observer." This is a departure from general practice, and it seems to the reviewer worthy of imitation by those who aim to reach correct results.

The author has added another name to the already extensive list,—"interstitial gingivitis." This does not seem to the reviewer to be any more appropriate than others have suggested to take the place originally given the disease. The reason given by the author for adopting this name is, "that the disorder is a local inflammatory condition of the gums." If this could be accepted, the name given would seem peculiarly appropriate, but clinical observation has failed to confirm this conclusion, and, in the opinion of the reviewer, pyorrhœa alveolaris holds but a limited relation to gingivitis; in fact, in the large majority of cases, the inflammatory condition of the gums, where this exists, is entirely of a secondary character. Hence the name, as a descriptive title, seems quite as much a failure as the many others sought to be fastened upon it. The old name, while equally defective, may, therefore, be allowed to retain its position, all the more that it is thoroughly understood.

The extensive series of microphotographs deserve especial praise, for they exhibit fairly well the results of the investigations made on lower animals, principally upon dogs. While this form of illustration is measurably defective in that the pictures are not always clear, they yet remove entirely the personal equation applicable to all drawings.

On page 46 the author heads a sub-chapter with "Do Glands exist in the Mucous and Peridental Membranes?" This important question, which Black first endeavored to answer, remains still a disputed question. The author summarizes an answer to his query by stating that "Black has attempted to demonstrate that glands exist in the structure, and that the cells last mentioned are glands. Black lays down as a *sine qua non* of a gland that there should be an opening to the surface. He has made an attempt to demonstrate such an outlet, but the figure does not show clearly that the glands empty into the duct or have an exit at the surface. These bodies, however, not only fail (like the ductless glands) in this particular, but in more important characteristics of glands. They do not have (as Robin and Magitot remark) a columnar or prismatic cell wall. It is not difficult to understand how epithelial cells are scattered in different shapes and sizes throughout the peridental membrane. Epithelial cells have the property of multiplying and developing in structures wherever located." While this, probably, does not settle the question, it is more nearly in accord with the views held by other histological workers.

Chapter V. is devoted to the consideration of "Uric Acid and Interstitial Gingivitis." After giving the history of this theory and quoting the opinions of its principal advocates, and also giving his own in opposition, which have been published heretofore, the author sums up his views as follows: "In an examination of nine hundred and fifty cases by different chemists at different periods, five to six per cent. give positive results as to uric acid by the chemic and microscopic examination. These results demonstrate conclusively that interstitial gingivitis is not due solely to uric acid; that uric acid when found is merely an expression of the uric acid diathesis and a coincidence, since it is not always present in the gums and tartar of patients attacked either by gout or the uric acid diathesis. In the six per cent. of cases there was nothing to show that uric acid was the cause of interstitial gingivitis, since the deposits were examined after the teeth had been removed. Any other irritation may have been the exciting cause. Uric acid acts, when at all, solely as a local irritant."

The question of a special bacterium for pyorrhœa alveolaris has claimed the attention of various investigators, the last on the list being Younger (INTERNATIONAL DENTAL JOURNAL). None of these can be said to have reached positive conclusions. Galippe claimed to have found in the pus of pyorrhœa an organism which when injected into a guinea-pig resulted in abscess. Miller cultivated a number of bacteria possessing pyogenic properties, but failed to find a specific organism. To aid in determining this question, the author had investigations made in the Columbus Memorial Laboratory in Chicago, and he gives in detail the results obtained. These he acknowledges are not conclusive standing alone, but, taken in connection with other "observations on this subject, are admissible." An attempt was made to produce this disease in dogs, with the result that "the pathological findings in these cases were not unlike inflammation and infection in other tissues. Similar results would, no doubt, have taken place if inoculation had been performed with pus from an abscess."

The general conclusions of the author do not vary materially from those generally accepted. On page 153 he gives a very clear statement as to the beginning and progress of pyorrhœa alveolaris. This covers very nearly the reviewer's view, and mainly eliminates constitutional conditions and places its origin where it properly belongs,—in local irritation. He, however, classes as predisposing influences, "syphilis, tuberculosis, mercurialism, plumbism, brass-poisoning, lithæmia, nephritis, gout, rheumatism, alcoholism, scurvy, nervous diseases, pregnancy, and old age."

It is with regret that, after such an able presentation of "interstitial gingivitis," the author should reach, in his chapter on "Treatment," such unsatisfactory conclusions. This is made evident by the following quotation: "The clinical history of interstitial gingivitis is essentially that of any other disease of the mucous membranes. The disorder responds quickly to treatment at its outset. Later its complications and the extent of structure involved render treatment very inefficacious, and always insure loss of the tooth." That such a statement should be made in view of the published accounts, by different operators, of complete cures, seems strange, indeed. The reviewer has repeatedly demonstrated that a restoration to health is possible in very advanced stages of the disease. Failure results from defective methods. That of the author may best be given in his own words:

"After the deposits have been fairly well removed, the gums may be syringed with hot water (Cravens) to remove all *débris*. The gums should be saturated with tincture of iodine (U. S. P.), or iodine and aconite, in the following manner: Have a number of wooden tooth-picks (Portuguese preferable) wound with cotton and kept in a little box. Saturate the cotton and paint the gum as far as it will go. . . . From three to five picks will be required. . . . In such cases a different massage brush is to be used. One is here required that will so lacerate and stimulate the gums as to relieve the congestions. . . . After two or three days the gums will contract and healthy circulation follow. . . . An astringent, stimulating, and general mouth-wash should be used in connection with the massage. The patient should return every other day for further treatment with the iodine, or iodine and aconite, used as before."

While all this is valuable as far as it goes, it is open to criticism, and it furnishes the key to the failure of the author to cure long-established cases of this disease.

With the exception of the concluding chapter, the reviewer is forced to regard this book as a very important contribution to the subject. That it has not definitely settled some obscure points in the etiology of pyorrhœa alveolaris must be acknowledged, but it may be asserted that it has fortified many clinical observations, and has thus advanced our knowledge many steps in advance of previous contributions.

The attention of the author is called to several errors in the spelling of proper names, notably those of Arkövy, Koecker, Nasmyth, and Bödecker.

With this exception, the book has been prepared with the usual care devoted to their publications by the S. S. White Dental Manufacturing Company.

A Review of Recent Legal Decisions affecting Physicians, Dentists, Druggists, and the Public Health, together with a Brief for the Prosecution of Unlicensed Practitioners of Medicine, Dentistry, or Pharmacy, etc. By W. A. Purrington, of the New York Bar, Counsel of the Dental Society of the State of New York and Lecturer of Medical and Dental Jurisprudence in the New York College of Dentistry, etc. E. B. Treat & Company, New York, 1899.

This compact volume of 105 pages is a timely production, and should be in the hands of every dentist. The time is not far distant in the past when to speak of the jurisprudence of dentistry would create a smile, but at the present time it is regarded of sufficient importance to claim the attention of some of the best legal minds. In proof of this we need only refer to the lectureships upon this subject established in the dental schools and to the men who fill them.

The author, in describing works in "legal medicine," etc., says of this: "The publishers . . . have therefore thought it worth while to issue its review of legal decisions during the past year as a separate pamphlet, adding to it a convenient brief of the law-points that usually arise in the prosecution of unlicensed practitioners of medicine, dentistry, and pharmacy."

The author evidently does not entertain the old-time opinion of a dentist, for he writes: "As the physician has ceased to be called a 'leech' and the surgeon a 'saw-bones,' so has the dentist ceased to be described by cheap wits as a 'tooth carpenter.'" In fact, the author places dentistry where it properly belongs, with other specialties of medicine.

In the chapter on "The Need of Examining Boards Illustrated" the author gives a long series of questions, to which are appended the answers in medicine and dentistry. It is impossible to see the value of this exposure of ignorance. It certainly does not furnish an argument for the appointment of State boards, for not a few instances are in evidence of ignorance emanating from that quarter.

Nothing is ever gained by such exhibits. They, doubtless, could be duplicated in every examination, collegiate or professional.

It is not possible to follow the author through the decisions given. The book should be kept for reference, and as it is of very moderate price, it comes within the reach of all, and every practitioner is interested in knowing how he stands before the law.

